# Possibilities of Using *Macrolepiota procera* in the Production of Prohealth Food and in Medicine

**DOI:** 10.1155/2022/5773275

**Published:** 2022-05-06

**Authors:** Iwona Adamska, Grzegorz Tokarczyk

**Affiliations:** Department of Fish, Plant and Gastronomy Technology, West Pomeranian University of Technology in Szczecin, Szczecin, Poland

## Abstract

Parasol mushroom (*Macrolepiota procera*) is a fungus that is often included in the menu of people looking for replacements for meat products and at the same time appreciating mushrooms. Its fruiting bodies are known for their delicate flavor and aroma. The aim of the publication was to analyze the latest information (mainly from 2015 to 2021) on the chemical composition of the *M. procera* fruiting bodies and their antioxidant properties. The data on other health-promoting properties and the possibilities of using these mushrooms in medicine were also compiled and summarized, taking into account their antibacterial, antioxidant, anti-inflammatory, regulatory, antidepressant, and anticancer effects. Moreover, the influence of various forms of processing and conservation of raw mushroom on its health-promoting properties was discussed. The possibilities of controlling the quality of both the raw material and the prepared dishes were also discussed. Such an opportunity is offered by the possibility of modifying the growing conditions, in particular, the appropriate selection of the substrate for mushroom cultivation and the deliberate enrichment of its composition with the selected substances, which will then be incorporated into the fungus organism.

## 1. Introduction

**Figure 1:**
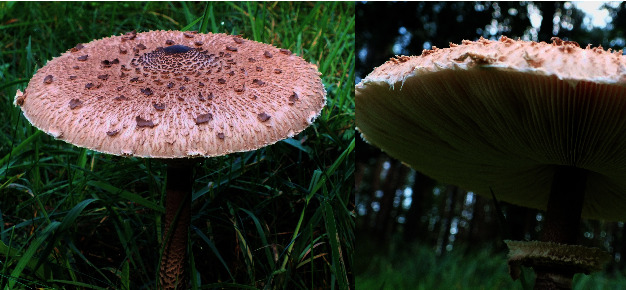
The fruiting bodies of the fungus *Macrolepiota procera*. The appearance of the mushroom from above and below.

*Macrolepiota procera* (Scop.) Singer is a fungus commonly called parasol mushroom, belonging to the family *Agaricaceae* (order *Agaricales*, division *Basidiomycota*, kingdom *Fungi*). This species has a whitish-brown, erected, and high (10-40 cm) stem, hollow in the center, with a large movable ring on it. The cap is large (10-30 cm in diameter), umbrella-shaped, in adult specimens; it takes a light (whitish) color with characteristic brown small patches. Under the cap, there is a lamellar hymenophore in the form of densely arranged gills [1, 2] (Figure 1).

Parasol mushrooms are eagerly picked and eaten by the inhabitants of many regions of the world. However, they are sometimes confused with food poisoning species. The aim of the study is to review the information published in 2015-2021 on the nutritional value and chemical composition of the *Macrolepiota procera* fruiting bodies, including the content of substances showing bioactive properties. The changes occurring in these mushrooms during the processing and preparation of dishes were also analyzed. Moreover, the paper discusses the possibilities of using these mushrooms in food production and medicine.

## 2. The Occurrence of *Macrolepiota procera* and the Possibility of Collecting Its Fruiting Bodies

### 2.1. Occurrence

This fungus is usually found in the wild: in its natural state, it occurs in coniferous forests, thickets, parks, meadows, and forest glades [1, 2], but the methods of cultivation are already known. The appropriate composition of the substrate has been developed to ensure high yields of the fruiting bodies. Currently, the mycelium of *M. procera* can be bought and inoculated in the garden, and the recommended substrate is shredded wood waste (branches, leaves, and chips). The starter packages contain mycelium placed in the composed substrate. Such a set should be placed in a hole dug in the ground, covered with mulch, and watered with water. The fruiting bodies usually appear only in the second year after inoculation [3]. Currently, a research is conducted to find the most optimal conditions for macroscale cultivation [4–7]. The quality of the substrate is important because its type and chemical composition always strongly influence the chemical composition of the mushroom fruiting bodies [8–11]. The methodology of culturing these mushrooms on laboratory and other types of media was also developed [4, 6, 7, 12]. It was found that the optimal conditions for their development were the temperature of about 30°C and the pH of the medium (potato dextrose agar media (PDA)) about 7. The largest colonies grew on the media in which the carbon source was maltose, and the nitrogen source glycine, with the carbon-to-nitrogen ratio being 10 : 1 and enrichment of the medium with 1% glucose [4].

*Macrolepiota procera* is found throughout Europe, in the eastern regions of North America, western Asia, and Japan, as well as in a few sites located in Australia, South America, Africa, and New Zealand [13, 14]. Information about this mushroom can also be found in a number of studies from many other parts of the world, e.g., India [15] or Mexico [16].

The production of cultivated mushrooms in the world continues to grow. The country with the most developed economy in this branch is China (Table 1) [17]. Edible mushrooms of the greatest economic importance in the world are the common mushroom (*Agaricus bisporus* (J.E. Lange) Imbach), shiitake (*Lentinula edodes* (Berk.) Pegler), and oyster mushroom (*Pleurotus ostreatus* (Jacq.) Quélet) [18]. *Macrolepiota procera* is one of the less popular species in terms of cultivation, despite the fact that it is a fungus with great potential both for the production of health-promoting food and the acquisition of substances with a healing effect. This is mainly due to the specific requirements of these mushrooms: the fruiting bodies develop best in shady places, near trees [19]. Commercial cultivation of these mushrooms is currently located in several regions of the world (as cited in [20]), including Korea [4] and Thailand (Kwon and Thatithatgoon 2004 after [21, 22]). However, there is no detailed information on the area under cultivation and the yields obtained.

*Macrolepiota procera* collected by mushroom pickers without experience can be easily confused with mushrooms belonging to the genus *Amanita* (especially *A. phalloides* and *A. pantherina*), *Chlorophyllum* (*C*. *rhacodes* (syn. *Macrolepiota rhacodes*) and *C. molybdites*), and *Lepiota* (*L*. *aspera*, *L*. *brunneoincarnata*, *L*. *helveola*, and *L. pseudolilacea*) [23]. It is advisable to collect only adult specimens. The young fruiting bodies of many mushroom species, including those with a lamellar hymenophore, have still undeveloped or poorly developed features that allow for certain and correct identification of the fungus. Therefore, in their case, it is easy to make a mistake; hence, they should not be picked. The mentioned species, unlike *Macrolepiota procera*, are poisonous fungi due to the content of amatoxins. There is a large differentiation of amatoxins within one genus of mushrooms, which is demonstrated by phylogenetic analysis carried out in close connection with chemical tests [24]. Amatoxins (amanitotoxins) are cyclic octapeptides containing sulfoxide and indole groups. They cause severe poisoning often leading to death. The toxin most strongly affecting the human body is *α*-amanitin (*α*-AMA). *α*-AMA is resistant to all forms of culinary processing: it does not deactivate even during frying and is not enzymatically deactivated. Approximately 30 minutes after consumption, it passes from the digestive system into the blood, and with it is distributed throughout the body. About 60% of *α*-AMA accumulates in the liver and causes severe damage to this organ [25, 26].

Due to the possibility of making a mistake, it is reasonable to have certificates confirming knowledge in the field of mycology and the ability to identify species in the case of people growing mushrooms and buying wild mushrooms for industrial purposes, including food. Such rules apply in the EU (European Union): a mushroom classifier course and a mushroom classifier course completed with state examinations allow you to obtain a certificate of a mushroom classifier or mushroom expert, respectively [27]. In some EU countries, the collection of mushrooms for private purposes is prohibited (Belgium) [28] or strictly limited (Germany [29, 30], the Netherlands [31], Great Britain [32], and Italy [33, 34]), often also additionally regulated by the local law (e.g., in Italy [33, 34], Germany [29, 30], and Switzerland [35]). These regulations mainly apply to public forests, while in the case of private forests, there is a very large variation depending on the area of Europe (state) and the ownership rights in force there [36]. However, in many Eastern European countries, the collection of mushrooms for private purposes is unlimited and uncontrolled, which can sometimes have negative effects (often mild poisoning, less often acute poisoning leading to loss of health or life) [37].

## 3. Nutritional Value of the Parasol Mushroom Fruiting Bodies

**Figure 2:**
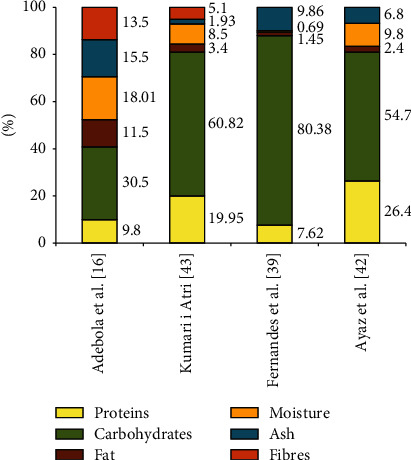
The percentage of the main nutrients in the fruiting bodies of *Macrolepiota procera* (in 100 g dw).

**Figure 3:**
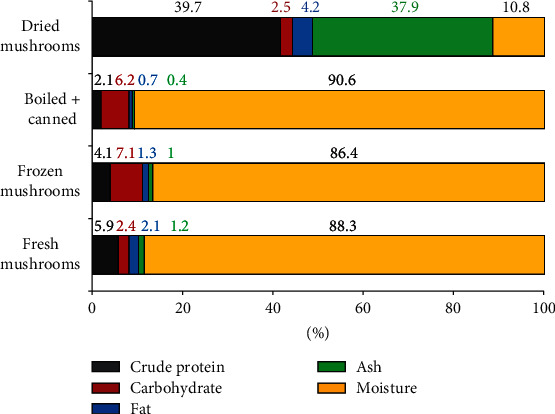
Composition of the fruiting bodies depending on the method of preservation in comparison with the fresh fruiting bodies (developed in: Aydin et al. [41]).

Water has the largest share in the fresh fruiting bodies of *Macrolepiota procera*. According to Mirończuk-Chodakowska and Witkowska [38], it constitutes 82.0-87.1% of the fresh mass of mushrooms, and according to Fernandes et al. [39], it is even more than 90%. These mushrooms are considered low in calories due to their low fat content [39–41], and at the same time, they are a rich source of carbohydrates, protein, and fiber [16, 40, 42, 43] (Figures 2 and 3; Table 2). *Macrolepiota procera* grown on liquid potato nutrient solution contains 29.7% dry weight (dw) of soluble proteins, of which albumins are the dominant group (18.7% dw). Globulins and prolamines have a smaller share: they account for 6.9% and 4.3% of dw, respectively [12]. In the composition of these mushrooms, the largest share is carbon (40.7% dw), nitrogen (6.3%), and hydrogen (5.9%) [44].

The mushroom fruiting bodies contain, depending on the species and environmental conditions in which they grew, 35-75% dw of carbohydrates. Most of them are indigestible polysaccharides and oligosaccharides (e.g., *β*-glucans, chitin, and trehalose) [45–47]. According to Barros et al. [48], the content of carbohydrates in the fruiting bodies of *Macrolepiota procera* was higher than the range given above and amounted to 80.38 g/100 g dw. These mushrooms contained 7.66 g/100 g dw in total sugars and only 0.32 g/100 g dw reducing sugars. The majority of sugars were mannitol (4.73 g/100 g dw), and a smaller part is trehalose (2.92 g/100 g dw) [48]. The content of total sugars in the fruiting bodies of this mushroom studied by Beluhan and Ranogajec [49] was 66.8 g/100 g dw, and a total content of soluble sugars and polyols is 24.4 g/100 g dw. Mannose and glucose had the highest share (11.1 g/100 g dw and 10.8 g/100 g dw, respectively), and the level of mannitol and trehalose was significantly lower (2.4 g/100 g dw and 0.1 g/100 g dw).

Fernandes et al. [50] found that 100 grams of the fresh fruiting bodies of *M. procera* contained a total of 70 g of carbohydrates, of which 15.7 g/100 g dw were sugars. Analysis of the composition of soluble sugars and polyols showed the presence of trehalose (9.1% dw), mannitol (5.2% dw), melezitose (1.24% dw), and fructose (0.06% dw) [50]. Insoluble and soluble fibers account for 30.5% dw and 3.4% dw of the *M. procera* fruiting bodies, respectively [47].

According to Mirończuk-Chodakowska and Witkowska [38] and Sari et al. [51], 10-11% dw of the fruiting bodies of *Macrolepiota* fungi are glucans. An advantage of beta-glucans over alpha-glucans has been found. This observation applies to both *M. procera* and *M. fuligonosa* (Barla) Bonn.

The calorific value of the fresh fruiting bodies, depending on the conditions in which they developed, was estimated at 52.2 to 57.5 kcal/100 g of fresh weight, respectively (Aydin et al. [41] and Alvarez-Parrilla et al. [52]), while the energy value of dried mushrooms was in the range of 206.6-353.7 kcal/100 g of dry weight [41, 43]. Calorific value of frozen mushrooms is similar to the value of the fruiting bodies in the fresh state (56.7 kcal/100 g of mass), and in the case of cooked and then preserved mushrooms, the lowest is 39.0 kcal/100 g [41].

Apart from the high nutritional value, the fruiting bodies of *Macrolepiota procera* are also characterized by significant functional properties and a high content of bioactive substances. They contain, among others, the following: free amino acids, fatty acids, and sterols (ergosterol). Among the free amino acids, proline, glutamic acid, serine, and alanine had the largest share [53, 54], the dominant carbohydrates were mannitol (4.73 ± 0.26 g/100 g of dry weight) and trehalose (2.92 ± 0.13 g/100 g of dry weight) [39], but the composition also included glycerol, glucose, and Lepiota [40].

The fruiting bodies of parasol mushrooms, like other species of fungi, contain large amounts of chitin, i.e., a linear polysaccharide composed of 2-acetylamino-2-deoxy-D-glucose units, between which there are *β*-glycosidic bonds in the 1,4 position (*β*-glucosamine polysaccharide) [40]. Although chitin plays a role similar to that of fiber in the diet, its presence is the reason why dishes containing mushrooms are considered difficult to digest and not recommended for children and the elderly [55]. However, this is a common view only in certain areas of Europe (Eastern Europe). In many other regions of the world, especially where there are problems with feeding people, mushrooms are recommended as an important component of the diet. Their consumption is recommended not only for adults but also for children. Mushrooms are regarded in these regions as a rich and valuable source of protein and mineral salts [56–58].

### 3.1. Fatty Acids

The share of saturated acids among fatty acids is 15.9%, and unsaturated fatty acids are dominant, accounting for 81.9%. Among the latter group, polyunsaturated acids have the largest share (62.4%). In these mushrooms, linoleic acid (62.4%) predominates among the fatty acids; oleic acid (17.4%) and palmitic acid (10.9%) have a smaller share [40]. Similar results concerning the share of individual acids and fatty acid groups were obtained in studies conducted in other regions of the world [39, 40, 53, 54].

### 3.2. Mineral Composition

The content of mineral salts in the fruiting bodies of various species of fungi has been the subject of many studies, among others, due to the ability of these organisms to accumulate heavy metals. It is known that the chemical composition of the substrate significantly affects the chemical composition of the mushroom fruiting bodies. Additionally, differences in the content of various elements in individual parts of the mushrooms (stems and caps) and the dependence of their content on the age of the fruiting body were observed.

The fruiting bodies of parasol mushroom are rich in sodium, potassium, magnesium, calcium, iron, manganese, zinc, and copper [16, 42, 43, 59, 60]. However, the content of different minerals varies depending on the part of the fruiting body analyzed. According to Radulescu et al. [2], a stipe contains significantly more iron than the caps, while the caps have far more copper than the stems. Similar results were obtained by Kojta et al. [60]; moreover, they found that the caps contained more K, Mg, Cd, Zn, Ag, Hg, Pb, and Rb than the stems, and there are more Al, Ba, Ca, Mn, Na, Sr, Co, Cr, and Ni in the stems than in the caps. These proportions also maintained when the fruiting bodies were obtained from contaminated areas. The content of minerals also depends on the age of the fruiting bodies: the highest amounts of P, Mg, Ca, and ash were found in young mushrooms compared to mature specimens [59].

### 3.3. Heavy Metals

On the basis of the bioaccumulation studies of the selected elements (Sr, Zn, Nb, Cs, Ba, Ce, Pb, Th, U, Nd, Al, V, Cr, Co, Ni, Cu, Zn, Ga, and Rb) in the mushroom fruiting bodies, it was found that the metal content mainly depended on the species and the amount of metal in the soil [61]. Among the substances accumulated in the mushroom fruiting bodies, the presence of various elements was found, including heavy metals and special purpose metals, metalloids, transition metals, alkali metals, alkaline earth metals, lanthanides and actinides, and nonmetallic elements [62].

Due to the features characterizing the thallus of parasol mushroom and its growth method, i.e., rapid development, strong branching of thin hyphae, and strong overgrowing of the substrate, this fungus quickly absorbs substances contained in the substrate and transports them to the fruiting bodies [62]. The fruiting bodies of this fungus strongly accumulate Ag, Rb, Cu, Zn, and Hg, among others, and are relatively rich in K, P, Zn, Cu, Fe, Al, and Mn [61]. The content of heavy metals clearly depends on the place of origin or cultivation of these mushrooms. During the research on the chemical composition of the fruiting bodies of *M. procera* from the Black Sea region (Turkey), a high content of Co (3.5 mg/kg dw), Cd (0.37 mg/kg dw), Ni (1.73 mg/kg dw), and Pb (2.58 mg/kg dw) was found [42]. Similarly, relatively high levels of some heavy metals have been observed in the fruiting bodies harvested in Nigeria (Cd: 0.29 mg l^−1^; Co: 0.18 mg l^−1^) [16].

The content of individual heavy metals differs depending on the analyzed part of the fruiting body of parasol mushroom. These mushrooms are particularly rich in Mg, P, Cu, Zn, and Mn. Caps, as most often the only parts of this mushroom intended for consumption, accumulate both greater amounts of the desired minerals (K, P, Mg, Zn, and Cu) and undesirable minerals in the diet (Rb, Ag, Pb, Hg, Cd, and Cr) than the stems. On the other hand, they have fewer essential ions of calcium, manganese, iron, and sodium, as well as the undesirable Ba, Sr, Al, Ni, and Co than in the stems. Particular attention should be paid to cadmium, which accumulates strongly in caps (even in the cap/stem ratio = 13). The comparison of the content of the examined elements with the guidelines of the EC directive showed that the content of lead and cadmium in the fruiting body exceeded the permissible standard [63]. Similar results were obtained by Gucia et al. [64]: caps of mushrooms from natural sites were characterized by a high concentration of K, Ag, Cu, Rg, P, Cd, Zn, Mg, Na, Al, Ca, Fe, and Mn, as well as strongly undesirable elements Cd, Hg, and Pb. Similar differences in the content of elements in different parts of the *M. procera* fruiting bodies were also observed by Kułdo et al. [65], but they also considered these mushrooms to be a rich source of Cu, Fe, K, Mn, and Zn. Similar results were obtained in other studies [16, 43, 60, 66–68]. Campos et al. [69] also determined the content of neodymium, thorium, and uranium in the fruiting bodies of this species, respectively, 5.43 *μ*g/g^−1^, 2.10 *μ*g/g^−1^, and 1.80 *μ*g/g^−1^.

Kosanić et al. [54] found that the content of all elements in the tested fruiting bodies of *M. procera* was within acceptable standards, except for cadmium. According to Kułdo et al. [65], apart from cadmium, the accumulation of mercury in the caps is also a big problem. Importantly, according to Falandysz et al. [70], even in caps of parasol mushrooms from unpolluted areas, the content of Cd, Hg, and Pb is elevated. According to Kojta et al. [60], consuming the fruiting bodies of *M. procera* once a week is safe for human health, but with more frequent consumption than once, we risk exceeding the permitted daily limits of Pb, Hg, Ag, and Cd provided in the diet.

Kojta et al. [60] found that mushrooms from natural (noncultivated) sites absorb greater amounts of Cd, Cu, K, Mg, Na, and Zn than is present in the substrate. According to Severoglu et al. [67], the content of heavy metals in mushrooms depends on soil pH and its organic composition, however, Kuziemska et al. [71] showed a low correlation of their content in fungal tissues with soil pH and high correlation with their content in the substrate. A lower accumulation of metals in the tissues of lamellar fungi was observed compared to tubular mushrooms, which was explained by the smaller amount of mycelium of lamellar fungi growing over the substrate, its faster growth, and shorter viability [72]. Mleczek et al. [66] also linked it to a specific type of mycelium growth of mushrooms with lamellar hymenophores: the exposure of their vegetative cell surface and a larger area of hyphae compared to tubular mushrooms. Sometimes, however, the differences in the content of certain elements in different species of mushrooms were very small. This has been observed, inter alia, in for Cd, Co, Hg, Ni, Pb, and Sr [61, 66, 69]. They can also accumulate elements from the radionuclide group, especially alpha-emitting radionuclides, e.g., ^210^Po and ^226^Ra [73, 74].

Mushrooms have the ability to bind (accumulate) metals due to the presence of specific proteins—metallothionein [75]. However, appropriate processing (cooking the caps without blanching them) allows to reduce the content of As, Cd, Hg, and Pb [70].

Heavy metals accumulate in the human body mainly in the liver, kidneys, and brain, although the presence of toxic substances has also been found in other tissues. Acute heavy metal poisoning often leads to death, but such forms of poisoning are rare. Frequent consumption of mushrooms increases the risk of chronic poisoning: although smaller amounts of heavy metals are introduced into the body at one time, but it takes longer and causes the accumulation of undesirable substances in the body [76].

Heavy metals are undesirable in food: it is forbidden to introduce into the production and trade of food products and raw materials containing too much of them. The permissible content of heavy metals in raw materials for the production of food and ready-made food products in the European Union is specified in the basic legal act: European Commission Regulation No. 1881/2006 of December 19, 2006 [77], and a number of regulations introducing changes to the basic act, including the most recent Regulation No. 1317/2021 of August 9, 2021 [78], and Regulation No. 1323/2021 of August 10, 2021 [79].

### 3.4. Bioactive Substances

The fruiting bodies of *M. procera* are a rich source of bioactive substances, including antioxidants (Table 2). They contain, among others, phenols, flavonoids, alkaloids, beta-carotene, lycopene [43], and saponins [16]. Mushrooms contain, among others, p-coumaric acid, p-hydroxybenzoic acid, protocatechuic acid, vanillic acid isomers, and cinnamic acid [39, 80–82]. Parasol mushrooms also contain a number of vitamins: they are rich in vitamin A (768.3 ± 0.2 in I.U.) [16], *α*-tocopherol (4.5 *μ*g/100 g dry weight (dw)) [50], and ascorbic acid (vitamin C; 0.77 ± 0.09 g/kg dw according Ayaz et al. [42], 0.098 ± 0.98 mg/g according Vishwakarma et al. [83], and 2.25 ± 0.14 − 2.45 ± 0.11 mg AAE/g dw according Erbiai et al. [82]). Moreover, their composition also includes malic acid (19.4 g/kg dw) and citric acid (40.86 g/kg dw) [42]. These ingredients have a health-promoting effect, including antioxidant.

Glucans are polysaccharides that build up and down the cell walls of bacteria, algae, plants, and fungi. There are two groups of glucans that differ in structure: alpha-glucans and beta-glucans. Due to the high bioactivity of beta-glucans, their sources are also sought among fungi, including noncultivated species. In Poland, the content of glucans was analyzed using two methods in 21 species of fungi and it was found that the fruiting bodies of parasol mushrooms contained the lowest glucans of all the mushrooms studied (11.4 ± 2.3 g/100 g dw). Among them, beta-glucans dominated (10.5 ± 0.3 g/100 g dw), and alpha-glucans constitute only a small part (0.9 ± 0.6 g/100 g dw). The *Pleurotus ostreatus* fruiting bodies contained the most glucans (45.9 ± 1.6 g/100 g dw) [40]. Glucans have an immunostimulating effect, increasing the body's resistance to infections caused by bacteria, fungi, and viruses by stimulating the activity of immune cells (including macrophages and monocytes). In addition, they support the body in preventing and fighting cancer (stimulate the activity of T lymphocytes) and inhibit the proliferation of cancer cells and accelerate their apoptosis [84–93]. Glucans supplied with food reduce the risk of cardiovascular disease by lowering the level of cholesterol in the blood [93, 94] as well as play a prebiotic role [95, 96].

The fruiting bodies of *M. procera* contain a lot of polyphenolic compounds, but their amount in dried mushrooms strongly depends on the drying method. The sun-dried fruiting bodies contained less polyphenols than freeze-drying (0.77 and 1.23 g% tannic acid, respectively) [97]. The content of these substances and flavonoids was higher in the aqueous extract than in the methanol extract [98], while comparing the different parts of the mushroom, it was found that there were more polyphenols in total in the caps than in the stems. The highest amount of total flavonoids was found in water extracts prepared from caps or in hydroalcoholic extracts prepared from stems. In these studies, very high peroxidase activity (1.5 U/g) and high catalase activity (9 *μ*mols H_2_O_2_/g/min) were also observed [2].

The total content of phenolic compounds in the fruiting bodies of parasol mushrooms according to the analysis carried out by the method of Slinkard and Slingleton [99] by Kosanić et al. [54] was 67.98 *μ*g PE/mg methanol extract and in the studies by Vishwakarma et al. [83] conducted according to Folin-Ciocalteu methods (23.89 ± 0.81 mg GAE/g dw). As a result of the comparison of this parameter characterizing 80% methanol extracts prepared from various species of mushrooms from Mexico, it was found that the fruiting bodies of *Macrolepiota* sp. contain these substances less than wild champignon and boletus, because there is only 100 mg CAE/100 g fresh weight (fw) (wild champignon: 308.3 mg CAE/100 g; boletus: 169.6 ± 26.7 mg CAE/100 g fw) [52], and according to Ayatar et al. [100], there are 45% more of them than in *Armillaria mellea* (*M. procera*: 36.25 ± 0.35 mg GAE/g extract; *A. mellea*: 20.87 mg GAE/g extract). According to the research on the composition of the methanol extract prepared from the parasol mushrooms fruiting bodies, it contains the most phenolic compounds (11.00 ± 0.87 mg/g dw), significantly less flavonoids (1.46 ± 0.04 mg/g dw), and alkaloids, *β*-carotene, and lycopene constitute a small percentage (0.048 ± 0.03 mg/g dw, 0.29 ± 0.07 *μ*g/g dw, and 0.07 ± 0 *μ*g/g dw, respectively) [43]. Small amounts of beta-carotene and traces of lycopene in methanol and water extract prepared from various parts of *M. procera* were also stated by Robaszkiewicz et al. [98]. In the studies of Vishwakarma et al. [83], the fruiting bodies of parasol mushrooms contained only 0.025 ± 0.61 *μ*g/mg of *β*-carotene and 0.650 ± 0.58 *μ*g/mg of lycopene. Ayatar et al. [100], comparing different species of mushrooms, found that the content of *β*-carotene in parasol mushrooms is 65% higher than that in *A. mellea* (*M. procera*: 0.091 ± 0.09 *μ*g/ml; *A. mellea*: 0.032 ± 0.04 *μ*g/ml), and the content of lycopene is half lower than in *A. mellea* (*M. procera*: 0.059 ± 0.02 *μ*g/ml; *A. mellea*: 0.11 ± 0.02 *μ*g/ml). Similar results from the comparison of these two species of fungi were obtained by Erbiai et al. [82]: *M. procera* from Morocco had more lycopene and *β*-carotene than *A. mellea*. However, in the case of mushrooms from Portugal, a reverse tendency was observed: the fruiting bodies of *A. mellea* were richer in these substances.

The presence of a number of phenolic acids in the fruiting bodies of *M. procera* was found: caffeic acid [101, 102], cinnamic acid [81, 82, 101–105], ferulic acid [82, 101], gallic acid [82, 101–103], gentisic acid [101, 103], *p*-coumaric acid [82, 101], *p*-hydroxybenzoic acid [82], protocatechuic acid [82, 101–103, 105, 106], syringic acid [82, 101, 103], tannic acid [101], and vanillic acid [81, 82, 101, 102], among others.

These mushrooms contain small amounts of phenolic compounds compared to other species (10.0 mg/g of extract) [101]. The presence of indole compounds is also of great importance for bioactivity. So far, 5-hydroksytryptophan, 5-methyltryptamine, indole, L-tryptophan, melatonin, and tryptamine have been isolated from parasol mushrooms. It is the presence of L-tryptophan and 5-hydroxytryptophan, as precursors of serotonin and melatonin, in addition to substances that easily penetrate the blood-brain barrier, that determine the importance of these mushrooms in the fight against depression [107]. According to the research of Fernandes et al. [50], the amount of 5-hydroxytryptophan in the fruiting bodies of this species is 10-22.9 mg/100 g dw.

An MpL (*Macrolepiota procera* lectin) similar in structure to ricin B with b-trefoil fold was isolated from the fruiting bodies of parasol mushrooms. It is a substance that protects the fruiting bodies of mushrooms against pests and parasites and has been shown to be toxic to the nematode *Caenorhabditis elegans* in laboratory tests [108]. The substances contained in the fruiting bodies of *Cantharellus cibarius* have a similar negative effect on the digestive system parasites [109, 110]. However, in the case of MpL, this substance, with positive results, was tested for its usefulness as a carrier of protein drugs, including anticancer drugs targeted at the interior of cells [108, 111]. Moreover, 12 triterpenes of the lanostane type (lepiotaprocerins marked with letters from A to L) were isolated and identified from ethanol extracts (90%) prepared from the dried and powdered *M. procera* fruiting bodies. Some of them showed high or medium bioactivity: substances A, B, C, D, E, and F were not cytotoxic to the cells of the selected tumor lines, unlike substances labeled G, H, I, J, K, and L (which will be discussed later in the publication). Moreover, lepiotaprocerins A, B, C, D, E, and F also had anti-inflammatory effects, and lepiotaprocerin D was the strongest in this respect [112].

Interesting, especially from the point of view of food producers, is the presence of fumaric acid in the fruiting bodies of parasol mushroom [113]. This substance is used as a preservative, acidity regulator, and antioxidant in food production processes (E297). It has a positive effect on extending the shelf life of products; however, its use is subject to the need to comply with the provisions on maximum doses in various types of products (from 1 g/kg in confectionery and instant products for the preparation of flavored teas and herbal infusions up to 4 g/kg in desserts and mixtures of dry powdered desserts). However, this acid is not allowed to be used in food products for infants and young children [114].

Mushrooms of the genus *Macrolepiota* can also be a source of substances that have not been described so far. An example is *M. neomastoidea*: in 2005, a new indole alkaloid called macrolepiotin was isolated from a methanol extract. However, the hopes for high antitumor activity in this substance have not been fulfilled: this compound did not show toxicity in laboratory tests against the selected cancer cell lines (A549 (non-small-cell lung carcinoma), HCT-15 (colon adenocarcinoma), SK-OV-3 (ovary malignant ascites), and SK-MEL-2 (skin melanoma)) [115].

## 4. Antioxidant Properties of *M. procera*

Studies on the composition of extracts obtained from many species of edible mushrooms confirm that many of them contain substances with antioxidant properties. Examples are mushrooms of the genus *Russula* [116] and *Macrolepiota procera* [117]. Often, however, wild mushrooms show higher antioxidant properties and a higher content of phenolic compounds than commercially grown mushrooms [52].

Many studies have demonstrated the antioxidant effect of methanol extract from the parasol mushroom fruiting bodies (Table 3), but its strength shows considerable differences: it is defined as at most medium [98] and sometimes as strongly reducing (DPPH radical scavenging IC_50_ = 311.40 *μ*g/ml) [54]. Ayatar et al. [100] showed the high antioxidant activity of methanol extract even at its low concentration. However, the antioxidant activity was also confirmed in extracts other than the methanolic one [54]. It was observed that this activity is higher in the case of water extracts than methanol extracts, which is influenced by the higher content of polyphenolic compounds and flavonoids in the water extracts [98]. In water extracts, depending on the method used, the activity was as follows: RSA (88.1 ± 2.1%), AAE (191.0 ± 43.5 mg · l^−1^), IC_50_ (0.95 mg), WES (14.37 ± 1.2 mg · ml^−1^), and TPC (2.4 ± 0.1 g · kg^−1^) [118].

There is a very high correlation (equal to 0.985) between the content of polyphenols in the tested mushrooms and the antioxidant activity of the obtained extract [44]. Popescu et al. [97] showed a very high content of polyphenolic compounds in extracts prepared from the preserved parasol mushroom fruiting bodies using the sun-drying method and the lyophilization method (0.766 and 1.232 g% tannic acid, respectively). The antioxidant nature of *M. procera* extract is related to the presence and composition of phenolic compounds. The extracts were characterized by the highest radical scavenging activity with the highest phenol content, which indicates an important role in this respect of phenolic hydroxyl groups in phenolic compounds [54].

The comparison of the antioxidant activity by the method of scavenging DPHH-free radicals of extracts from various species of mushrooms showed that an extract from *M. procera* has a stronger effect in this respect than the extract from *Armillaria mellea*(IC_50_ = 0.191 mg/ml and IC_50_ = 1.190 mg/ml; control BHT IC_50_ = 0.096 mg/ml, respectively). The authors of the research explained it by the dependence on the total phenolic compounds content and the amount of beta carotene and lycopene in the fruiting bodies [100]. However, in the studies by Alvarez-Parrilla et al. [52], the antioxidant activity of methanol extract determined by the FRAP method was lower than that of boletus and wild champignon (parasol mushroom: 1.8 mmol FE^2+^/100 g fw; boletus: approx. 3.20 mmol FE^2+^/100 g fw; wild champignon: 4.49 mmol FE^2+^/100 g fw), also lower than for strawberries, but higher than for peaches. Also in these studies, a very high correlation was found between the total amount of phenols and the antioxidant activity (0.9721). This activity depended on the type of phenolic compounds present in the mushrooms. Hussein et al. [119] showed a low content of phenolic compounds in methanol extracts compared to other fungi (136.21 ± 0.98 mg GAE/100 g). They contained very small amounts of *β*-carotene (11.57 ± 2.39 mg 100 g^−1^) and lycopene (5.37 ± 0.55 mg 100 g^−1^). These studies also showed a very low overall content of flavonoids (8.66 ± 1.08 mg QE 100 g^−1^), as well as low DPPH activity (%) at the level of 65.41, and at the same time high chelation activity (91.45% of FE^2+^). Cinnamic acid, a substance showing antioxidant activity, was also isolated from the fruiting bodies of *M. procera* [39, 80, 81], while fructogalactan (PS II) was isolated from an aqueous extract prepared from the fruiting bodies of *M. dolichaula* (a species related to *M. procera*). This substance showed an antioxidant effect in tests [120].

## 5. The Fruiting Bodies of *M. procera* in Food Production

### 5.1. Preparation of the Fruiting Bodies for Consumption

Two rules should be followed for a safe consumption of *M. procera*: the fruiting bodies should be cleaned and heat treated before consumption (eaten raw may cause indigestion) [65, 121]. Only caps should be used to prepare a meal, because the stems are hard, hollow, and unpalatable, and eating them can also cause digestive problems.

For food purposes, it would be best to use cultivated mushrooms, i.e., mushrooms produced under controlled conditions. In this case, through the appropriate selection of substrates in the substrate, we can regulate and control the composition of the fruiting bodies. However, the production of these mushrooms is not yet developed enough to completely replace the collection of the fruiting bodies from forest and shrub communities. If we use mushrooms from natural sites in food production, according to EU legislation, they must come from sources (companies) that issue a certificate confirming their species affiliation [26]. It would also be important to study the chemical composition of the fruiting bodies, because fungi accumulate many substances present in the substrate and in the air (often these are pollutants, e.g., heavy metals). *Macrolepiota procera* has such a tendency to accumulate. Hence, Kojta et al. [60] and Falandysz et al. [63] found that the consumption of the fruiting bodies of this mushroom once a week is safe, while more frequent consumption is not recommended.

According to the rules adopted in the EU, whole mushrooms (stem and cap) should go to the sale and production of food, which guarantees that there will be no mistake. When purchasing dried mushrooms, the packaging should be tight and carefully labeled (necessary information: species name of the mushroom, net weight, certificate number, date of harvest, use by date, producer, nutritional values for 100 g of the product, and information on allergens); moreover, they must contain the same number of caps and stalks [26].

The fruiting bodies of parasol mushroom are considered an alternative to meat: when properly prepared, their taste is very similar to that of meat chops. These mushrooms are recommended for people on a low-fat diet [16], and due to the rich chemical composition and high biological activity of the substances they contain, they can be treated as functional food or nutraceuticals [39]. Similar conclusions were also drawn by Nowak and colleagues [122], who analyzed the importance of polysaccharides contained in the fruiting bodies of wild mushrooms (including *M. procera*) for stimulating the growth of the intestinal bacteria *Lactobacillus acidophilus* and *L. rhamnosus*. Although the ethanol extract from *Macrolepiota procera* contains only 15.7% polysaccharides, these substances were found to be more effective than the prebiotics available on the market (inulin or fructooligosaccharides), because they do not undergo hydrolysis when passing through the stomach (the degree of hydrolysis in gastric juice at pH 1 and equal to 5 is very low and amounts to 0.98% and 0.72%, respectively). The polysaccharides reach the colon unchanged and stimulate the growth of colonies of beneficial bacteria there (in the case of *Lactobacillus acidophilus*, they have a weak effect (18.88%) and much stronger in relation to *L. rhamnosus* 1 (27.9%)). This was considered to be an indicator of the high value of the polysaccharides contained in the parasol mushroom fruiting bodies for the production of functional food and nutraceuticals. Ćirić et al. [123] had a similar opinion on the use of these mushrooms in the production of functional food.

The influence of prebiotics on human health is extremely beneficial and multidirectional. They alleviate intestinal disorders and inflammation of the large intestine, improve intestinal peristalsis, and regulate the absorption of phosphorus and calcium. In addition, they reduce the absorption of lipids, which leads to a reduction in obesity [124–127]. Prebiotics also improve the functioning of the circulatory system [128]. Their use in food production offers many possibilities due to the fact that they have no influence on the product matrix. According to Sip and Grajek [129], they can be incorporated into various products without harming their sensory values, bioactive properties, and nutritional values, including products of the confectionery industry (including chocolate products, cakes and pastries, or cake masses) and bakery industry, as well as for beverages, food concentrates, soups, and convenience food.

Hussein et al. [119] recognized the fruiting bodies of *M. procera* as a valuable source of natural bioactive substances, showing, inter alia, antioxidant activity. They suggested that there are very large possibilities of using their high activity for food purposes, which is related to the high availability of this raw material and high acceptance by the society.

### 5.2. Effect of Treatment on the Chemical Composition and Antioxidant Properties

The chemical composition and especially the content of minerals absorbed by mushrooms from the substrate change during the processing of raw mushrooms. The size of these changes is influenced by the method and temperature of mushroom treatment. This is evident in the mercury content. Both cooking, blanching, and slicing combined with freezing reduce the content of this element in mushroom (while during the latter form of processing, the Hg level drops by as much as 35%, and as a result of blanching sliced mushrooms, the level drops by 15%). Interestingly, neither the processing time nor the type of water significantly influenced the level of this element: no differences were found between the batches subjected to a 5-minute or 15-minute blanching and between the samples prepared with potable or deionized water. Also, the pickling process did not affect the mercury level in the fruiting bodies [130].

Thermal treatment of the parasol mushroom fruiting bodies also causes a decrease in the content of indole compounds compared to the fresh fruiting bodies. These compounds are very sensitive to high temperature; its increase causes a partial decrease in the amount of some of these substances. In the treated mushrooms, only the tryptamine level increased and the indole level remained unchanged, while the L-tryptophan, 5-methyltryptamine, and 5-hydroxytryptophan content decreased and melatonin has completely disappeared [107].

The amount of antioxidants in mushrooms depends on the age of the fruiting bodies. The young fruiting bodies are usually the most valuable. In the mature fruiting bodies of *Lactarius*, the content of antioxidant compounds (phenols, ascorbic acid, and beta-carotene) was significantly lower than in the young fruiting bodies, which is related to the differences in the intensity of defense mechanisms in the fruiting bodies of different ages and the aging process of the mature fruiting bodies [131]. In addition, the age of the mushrooms is related to the length of time they must be heat treated. The older fruiting bodies, due to the stronger structure of the cell walls, require longer thermal processes. The temperature used during thermal treatment also significantly influences the antioxidant activity of mushrooms. In the case of processes carried out at high temperature (e.g., cooking), the level of antioxidants contained in the tissues of *M. procera* significantly decreases, which is caused by the degradation of the polyphenol structure. As a result, the antioxidant activity of dishes containing the fruiting bodies of this mushroom is reduced [48, 131]. During such a procedure, as a result of cell wall disruption, polyphenolic and flavonoid compounds are more easily released from the cells in comparison to the untreated raw material, and they flow out of the fungal tissues [131, 132]. However, when processing at lower temperatures (e.g., heating), the concentration of polyphenols often increases (e.g., in dried mushrooms) [48, 106], because new compounds with antioxidant properties are formed under the influence of heat and thermal treatment [131, 132].

### 5.3. Effect of the Method of Preservation on the Chemical Composition of the Fruiting Bodies

The processing of parasol mushrooms is essential for preparing the fruiting bodies for consumption. In addition, these are mushrooms, the fruiting bodies of which can easily spoil due to the high water content and not too dense flesh, so treatments are necessary to extend its shelf life. During traditional processing, chemical changes take place in it; therefore, while looking for more effective methods of preserving the quality of the fruiting bodies, the possibilities of irradiating the fruiting bodies were adopted. The content and composition of organic acids and phenolic compounds in irradiated mushrooms and the dried, frozen, and fresh fruiting bodies were compared. Irradiation as a method of preservation is characterized by high safety and ensures an appropriate level of hygiene of the preserved raw material, as well as ensures its high sensory quality, and also requires little financial expenditure. Stronger irradiation did not cause significant changes in the quality of the raw material, while traditional preservation methods resulted in greater losses of organic acids and total phenolic acids than the applied strong irradiation. The combination of irradiation and other preservatives resulted in fewer chemical changes than those following normal preservation processes: drying and freezing reduced the content of total phenolic acids, total organic acids and protocatechuic acid, p-hydroxybenzoic acid, p-coumaric acid, and mallic acid. In dried mushrooms, there was more quinic acid and fumaric acid than in fresh mushrooms, and the content of citric acid and cinnamic acid was highest in frozen mushrooms. As a result of the research, irradiation was recognized as an effective complementary technology that reduces the negative effects of dehydration and freezing of the mushroom's fruiting bodies. Irradiation with gamma rays (1 kGy) showed higher amounts of malic acid, citric acid, and cinnamic acid than in nonirradiated mushrooms (0 kGy), while the content of oxalic acid, quinic acid, protocatechuic acid, and p-hydroxybenzoic acid in mushrooms exposed to gamma radiation slightly decreased [133].

### 5.4. Noncommercial Use of the *M. procera* Fruiting Bodies

**Figure 4:**
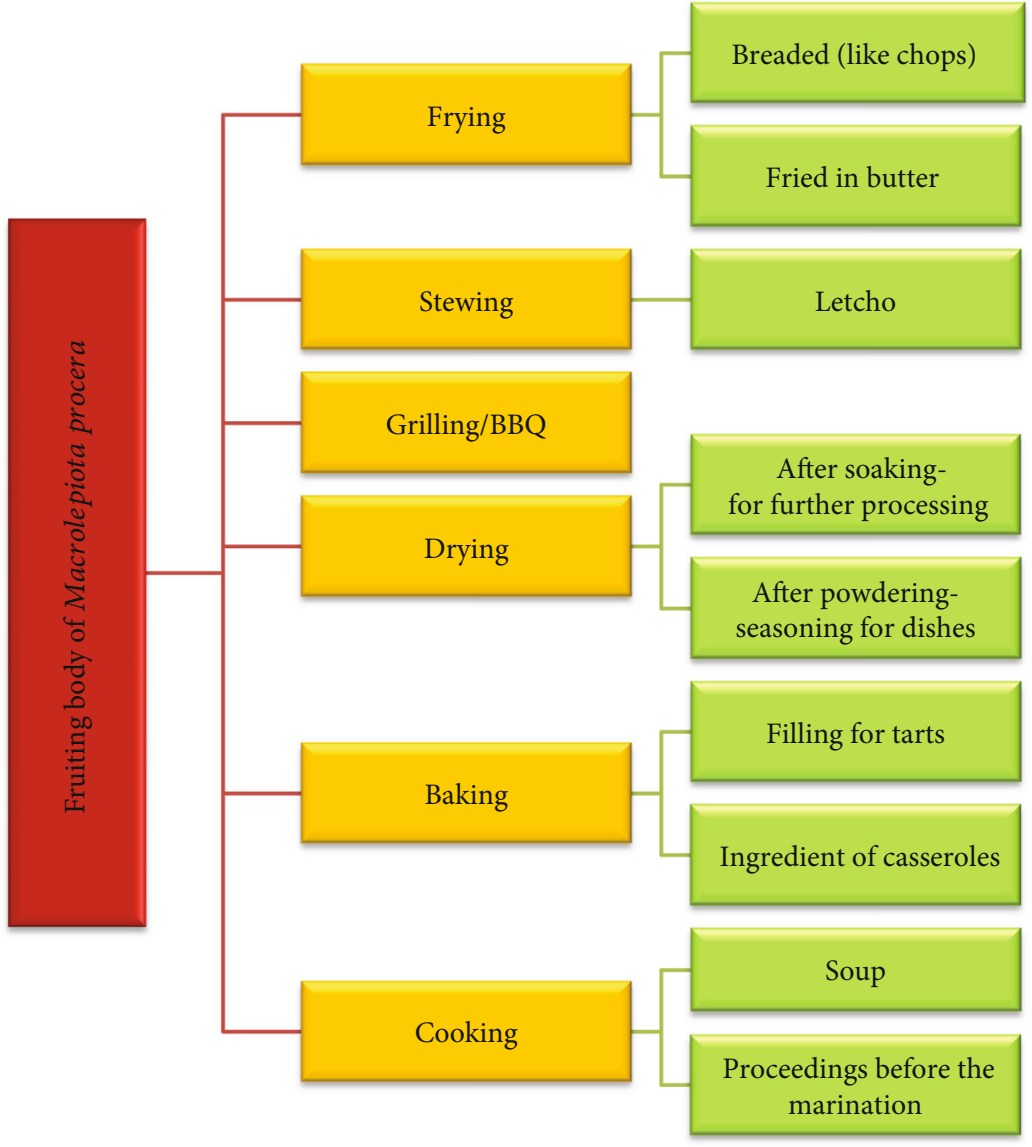
The use of the *Macrolepiota procera* fruiting bodies for consumption.

The fruiting bodies of parasol mushrooms are considered edible and very tasty [4, 6, 7] and even unique or extremely tasty [42, 60]. Their taste and smell are assessed as pleasant but not very strong [1]. There are many ways to prepare these fruiting bodies for home consumption (Figure 4). Most often, caps are prepared as cutlets: they are coated in breadcrumbs or flour and then fried in hot oil or butter. In this form, they are consumed, among others, in France, Italy, Ukraine, and Poland. Less often, this mushroom is used to prepare a soup (goulash or tripe in Poland), treat or tart filling, or eat it fried in butter, grilled, baked with eggs, or stuffed and baked. In some parts of the world, after drying, caps are ground and used as seasoning for soups, or dried caps are used to prepare dishes after soaking in water [70]. However, the drying process increases the nutrient content associated with the loss of water during the drying process. The calorific value of a 100-gram portion of such raw material also increases [41]. Caps of *M. procera* should not be blanched before they are properly processed, as this process favors the loss of minerals that leak into the water [70], but according to Aydin et al. [41], the best way to preserve the parasol mushroom fruiting bodies is to freeze them. This allows you to maintain the content of basic nutrients in amounts closest to the composition of substances contained in the fresh fruiting bodies.

## 6. The Importance of Bioactive Substances Found in *M. procera* for Human Health

**Figure 5:**
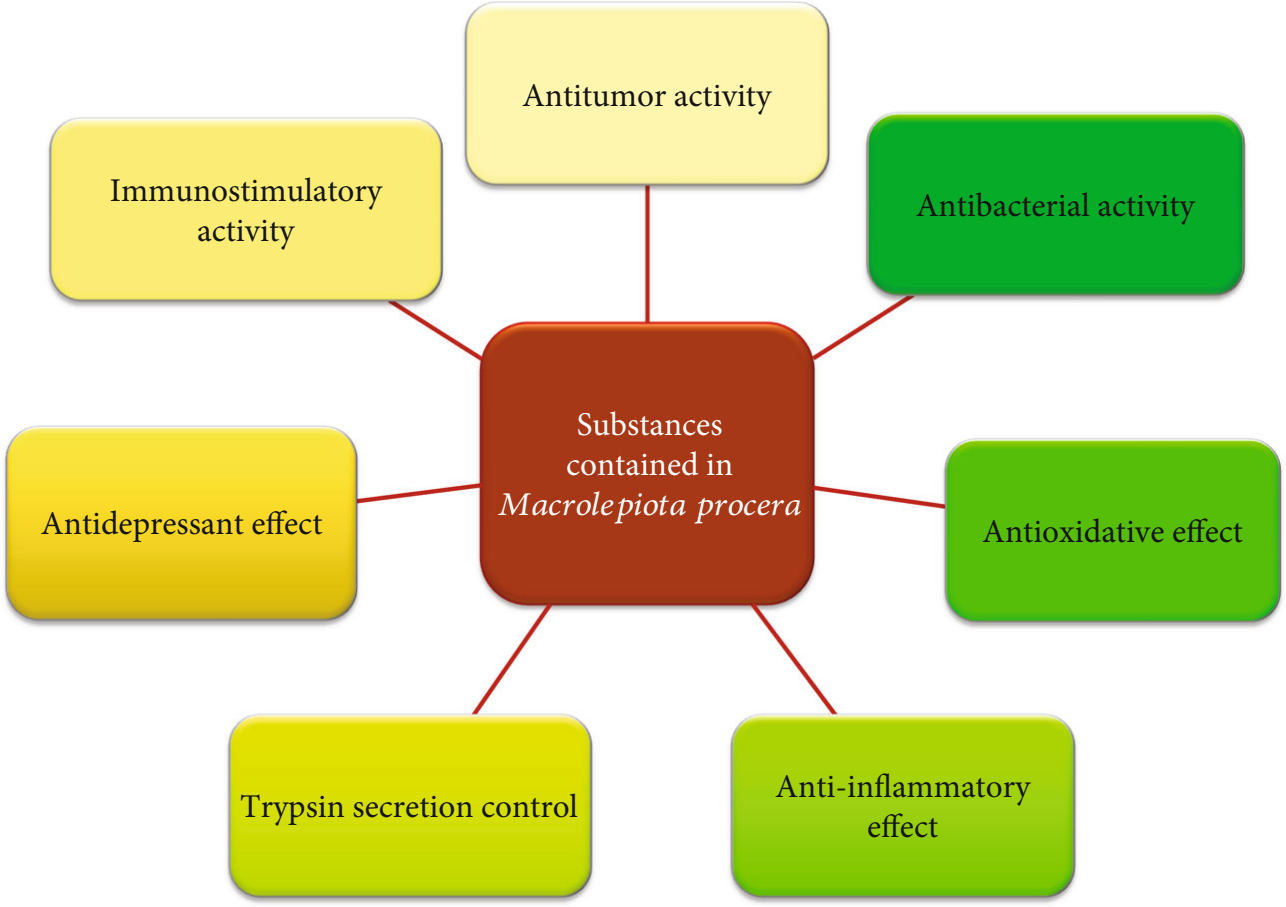
Bioactive effect of *Macrolepiota procera.*

The bioactivity of the substances contained in mushrooms is related to several most important aspects: the antibacterial, anti-inflammatory, anticancer, and antioxidant effects on the human body (Tables 4 and 5). In addition, it has been shown that the fruiting bodies of *M. procera* also have an immunostimulating effect and regulate the functioning of the digestive system (specifically the pancreas). One cannot ignore the antidepressant and prebiotic effects on the human body (Figure 5). Shim et al. [4] showed that the fruiting bodies of this mushroom exhibit healing properties. Similarly, Adebola and colleagues [16] recognized the high healing potential of *Macrolepiota procera* mushrooms and recognized its usefulness in the pharmaceutical industry and medicine.

### 6.1. Antioxidant Effect

The antioxidant activity of fungi is related to the content of a number of substances, including phenolic compounds (quercetin and catechin), phenolic acids (coumaric, caffeic, and gallic acids, ferulic, p-hydroxybenzoic, and homogentisic acids), and flavonoids, including catechin, vitexin, luteolin, kaempferol, naringenin, apigenin, quercetin, and rutin [134, 135]. Tocopherols, ascorbic acid, and carotenoids (including beta-carotene and lycopene) and vitamins also play an important role here. These substances are present, inter alia, in the parasol mushroom fruiting bodies. They catch free radicals, which slows down the negative changes occurring under the influence of oxidative stress, i.e., premature aging of cells and changes in their genetic system. Thus, they reduce the likelihood of neoplastic diseases. Oxidative stress also contributes to the development of diseases related to the malfunctioning of the circulatory and nervous systems [136–140].

Phenolic compounds and carotenoids have a positive multidirectional effect on the human body, but their main activity is antioxidant. Phenolic compounds also have anticancer, anti-inflammatory, antibacterial, and protective properties against the circulatory, nervous, digestive, and endocrine systems [141], and carotenoids have antitumor, immunostimulating, and protective properties of the cardiovascular system and the eyes. In addition, they are anti-inflammatory, reduce lipid accumulation and insulin resistance, and prevent liver damage [138, 140, 142, 143].

Antioxidant activity is also important for increasing the shelf life of food products due to the delay in the aging/rancidity of some ingredients, color changes, and a decrease in nutritional value. The substances contained in mushrooms can be used as a natural inhibitor of oxidation processes or as a synergist and play both a preventive and interventional role (Yanishlieva-Maslarova 2001 after [144]).

Antitumor activity of these fungi is related to the presence of polysaccharides and lectins in them [54, 139]; however, the role of cadmium as a component that has a toxic effect on neoplastic cells is also not excluded. The cytotoxic activity of methanol extracts obtained from the *M. procera* fruiting bodies has been demonstrated against HeLa human epithelial carcinoma cells, A549 human lung carcinoma, and LS174 human colon carcinoma cells. There was a clear effect on all tested cell types; however, compared to the other analyzed fungal species (*Lactarius deliciosus*), they had a stronger effect on A549 and LS174 than on HeLa. They also influenced the viability of healthy cells [54]. Interesting and promising is the fact that the water extract of parasol mushrooms inhibited colon cancer metastasis caused by colon 26-M3.1 cells [145].

Research on the antitumor activity of methanol extracts from *Macrolepiota* fungi was also carried out by Ćirić and colleagues [123]. However, they showed that *M. procera* did not negatively affect the selected tumor cell lines (breast carcinoma MCF-7, non-small-cell lung cancer NCI-H460, cervical carcinoma HeLa, and hepatocellular carcinoma HepG2). The authors of the studies noted, however, that their results contradict the results obtained by Arora et al. [146], which proved the antitumor activity of *M. procera* ethanol extract on MCF-7, colon cancer cells COLO-205, and kidney cancer cells ACHN and a very strong antiproliferative effect of water extract on COLO-205.

12 triterpenes of the lanostane type were also isolated and identified from ethanol extracts of the dried and powdered parasol mushroom fruiting bodies: lepiotaprocerin A-L. Their antitumor activity was tested against the selected human tumor cell lines (human myeloid leukemia HL-60, human intracellular carcinoma SMMC-7721, lung cancer A-549, breast cancer MCF7, and colon cancer SW-480). Lepiotaprocerins A and F had the ability to strongly inhibit the production of nitric oxide (IC_50_ 17.9-34.9 *μ*M). Lepiotaprocerins G, H, I, J, K, and L showed different cytotoxic potencies in relation to cancer cell lines, and compounds from lepiotaprocerin A to lepiotaprocerin F were inactive in this respect. Lepiotaprocerin I also showed antituberculosis activity (it significantly inhibited the growth of *Mycobacterium tuberculosis* colonies). The studies showed significant toxicity of lepiotaprocerins G, H, and L and moderate toxicity of lepiotaprocerins I, J, and K [112]. On the other hand, macrolepiotin (indole alkaloid) isolated from a related species (*Macrolepiota neomastoidea*) showed no toxicity towards the selected cancer cells [115].

### 6.2. Antimicrobial and Antifungal Activity

In order to demonstrate the potential of mushrooms to inhibit the growth of pathogenic bacteria and fungi, the most common tests are the effects of acetone, chloroform, ethanol, methanol, or water extracts on strains of the selected species, determining the selected growth parameters. Extracts obtained from many species of edible mushrooms in such tests showed an inhibitory effect on the development of pathogens [54, 100, 147–153]. Such studies were also carried out for the fruiting bodies of *M. procera*. The antibacterial activity of parasol mushroom is mainly influenced by the presence of terpenoids and phenolic compounds in these fruiting bodies [139]. Moreover, Kosanić et al. [54] suggest that this activity may also be related to the presence of cadmium in the tissues of the fungus (and the extracts prepared from them). According to them, it is this element that can have a toxic effect on microorganisms, causing an antibacterial effect. These scientists demonstrated in their research the antibacterial activity of a methanol extract prepared from parasol mushrooms against 3 species of bacteria (*Bacillus cereus*, *B. subtilis*, and *Proteus mirabilis*), while *Escherichia coli* and *Staphylococcus aureus* showed resistance. However, the effect of the *M. procera* extract was weaker than that of the other fungus species included in the study (*Lactarius deliciosus*) [54]. Extracts obtained from *M. procera* had a stronger effect on gram positive (G+) bacteria than gram negative (G-) [54, 150], which is due to the inhibition of the synthesis of cell walls, proteins, or nucleic acids in bacterial cells. However, these extracts have been shown to be less effective than the standard antibiotic given for bacterial infections (streptomycin). The research showed that the studied mushroom extracts had a stronger effect on the biology of bacteria than on fungi, which is related to the completely different structure and composition of the cell wall in these two groups of organisms. This aspect, moreover, makes bacteria more susceptible to antibiotics than fungi. However, when examining the effect of *M. procera* extract on the selected species of fungi, it was found that they inhibit the development of *Alternaria alternata*, *Aspergillus niger*, *Candida albicans*, *Cladosporium cladosporioides*, *Fusarium oxysporum*, *Penicillium expansum*, and *Trichoderma viride*. Fungi *Aspergillus flavus* and *Mucor mucedo* showed the highest resistance. However, the effect of the extract is much weaker than that of the preparation used in infections and diseases of fungal origin—ketoconazole [54].

Ćirić and his team [123] also tested the effectiveness of methanol extracts from 3 species of *Macrolepiota* mushrooms against the selected strains of microorganisms (bacteria: *Bacillus cereus*, *Enterobacter cloacae* ATCC 35030, *Escherichia coli* ATCC 35210, *E. coli* H2b, *Pseudomonas aeruginosa* ATCC 27853, *P. aeruginosa* IBRS P001, *Staphylococcus aureus* ATCC 6538, and *S. aureus* MRSA; fungi: *Aspergillus fumigatus* ATCC 9197, *A. niger* ATCC6275, *A. ochraceus* ATCC 12066, *A. versicolor* ATCC 11730, *Penicillium funiculosum* ATCC 10509, *P. ochrochloron* ATCC 9112, *P. verrucosum* var. *cyclopium*, and *Trichoderma viride* IAM 5061). The authors of the research concluded that the substances contained in these fungi have an antibacterial effect, but they affect individual bacterial strains with varying strength. During the research, it was shown that *M. procera* showed the weakest antibacterial effect compared to the other representatives of the genus *Macrolepiota* (*M. mastoidea* and *M. rhacodes*). On the other hand, parasol mushroom extract had a stronger antifungal activity than *M. rhacodes*. It is promising that extracts from all *Macrolepiota* species, including *M. procera*, tested against antibiotic-resistant bacteria species (*E. coli*, *P. aeruginosa*, and *S. aureus*) were more potent than ampicillin.

The effectiveness of water extracts from the fruiting bodies of *M. procera* has also been demonstrated against gram-negative (G-) aerobic *Pseudomonas aeruginosa* bacilli. Their inhibitory effect was found against *P. aeruginosa* 44 strain obtained from the cattle lungs and *P. aeruginosa* 119 strain obtained from the human lungs. It was found that the antimicrobial activity of parasol mushroom extract positively correlated with total phenolic content [118]. The inhibitory effect of *M. procera* extracts against *Escherichia coli* ATCC 25922 and *Klebsiella pneumoniae* ATCC 13883 was also confirmed, while bacteria *Acinetobacter haemolyticus* ATCC 19002, *Enterococcus faecalis* ATCC 29212, *Pseudomonas aeruginosa* ATCC 27853, *Salmonella typhimurium* ATCC 14028, and *Staphylococcus aureus* ATCC 25923 showed resistance [44]. Ayatar et al. [100] proved, however, that methanol extracts prepared from *M. procera* exhibit antimicrobial activity, although weak, against *E. faecalis* ATCC 29212 and *K. pneumoniae* ATCC 13883, while they do not negatively affect the colonies of *Bacillus cereus* NRRL B-3711, *B. subtilis* ATCC 6633, *Enterococcus hirae* ATCC 9790, *Escherichia coli* ATCC 25922, *E. coli* ATCC 35218, *Proteus vulgaris* RSKK 96029, *Pseudomonas aeruginosa* ATCC 27853, *Salmonella typhimurium* ATCC 14028, *Staphylococcus aureus* ATCC 25923, and fungi *Candida albicans* ATCC 10231, *C. krusei* ATCC 6258, and *C. tropicalis* Y-12968.

Antimicrobial activity was also demonstrated by the chloroform-acetone extract from the fermentation fluid left over from the cultivation of *Macrolepiota konradii*, a mushroom related to *M. procera*. Its effectiveness has been proven both against G+ bacteria (it inhibited the development of *Staphylococcus aureus*, *S. epidermidis*, and *Micrococcus luteus*) and G- (*Escherichia coli*). However, this extract did not show any antifungal activity [154].

### 6.3. Anti-Inflammatory Effect

The conducted studies on the effect of methanol extracts obtained from the fruiting bodies of 38 selected mushrooms on the course of inflammation previously induced by the administration of 12-O-tetradecanoylphorbol-13-acetate (TPA) to the ear of mice showed that some species of fungi showed a strong inhibitory effect on the development of inflammation. TPA present in croton oil is considered to be a factor promoting the formation of neoplastic changes. After its administration, swelling appeared on the tested part of the mouse's body, which was then applied with mushroom extracts. The extracts obtained from the fruiting bodies of *M. procera* showed an inhibitory effect on inflammation, but it was very small. At the same time, these extracts were not very effective in preventing the formation of neoplastic changes compared to other species included in the research [155].

### 6.4. Regulating Activity

Macrospin, a substance isolated from the fruiting bodies of *M. procera*, is a very strong trypsin inhibitor and a weak chymotrypsin inhibitor. It is very similar in structure to cnispin, a trypsin inhibitor found in *Clitocybe nebularis*. Macrospin is resistant to high temperatures and extreme pH values (it maintains its activity even at a temperature of 80°C and at pH 2 and pH 11). It has been found to be an effective agent in the regulation of trypsin secretion. In studies, it did not show any inhibitory effect on other serine proteases (thrombin, kallikrein, elastase, and subtilisin), cysteine protease papain, or pepsin of aspartic protease [156].

### 6.5. Immunostimulating Effect

Fucogalactan (PS II) was isolated from aqueous extracts prepared from the fruiting bodies of the fungus *Macrolepiota* (*M*. *dolichaula*, a species related to *M. procera*). This substance in laboratory tests activated macrophages *in vitro* and activated splenocyte and thymocyte in murine cell cultures. Its simple isolation from fungal tissues and the ease of possible administration for medical purposes or use in other production branches are associated with its solubility in water [120].

### 6.6. Antidepressant Effect

Mushrooms are a valuable source of indole derivatives (L-tryptophan, 5-hydroxytryptophan, tryptamine, serotonin, and melatonin), which are neurotransmitters and their precursors [157]. It is recognized that mushrooms do not lose their value (resource of indole compounds) even after heat treatment [107, 158]. Parasol mushrooms contain large amounts of 5-hydroxytryptophan, which is a precursor to serotonin and methionine, but the highest levels of it were found in the fresh fruiting bodies (22.94 mg/100 g dw). During cooking, its quantity decreased: the extract of the cooked fruiting bodies contained only half of that of raw mushrooms (10.11 mg/100 g dw). Similarly with melatonin, there was more of it in the fresh fruiting body extract than in the processed mushroom extract [157]. Indole compounds show, in addition to the antidepressant effect, also antioxidant, anticancer, and antiaging effects. Moreover, they act as regulators of the circadian cycle and influence the mechanisms of blood coagulation [107]. In addition, the fruiting bodies of this mushroom also contain one of the microelements supporting the antidepressant effect—zinc [157]. Along with the growing awareness of societies about the consequences of depression, products and dishes prepared based on the *M. procera* fruiting bodies seem to be a good supplement to the diet, especially in the case of people prone to depression or periodic mood deterioration.

## 7. Conclusions

Health-promoting food should fulfill two main functions: nutritional and exert a beneficial (prohealth) effect on the body. This influence should be documented by a scientific research. Prohealth food should have a preventive effect on various ailments, support healing processes, improve the health of the body, or inhibit the development of adverse changes, e.g., aging processes. In addition, it must be in the form of a food product made of natural food ingredients, with the possibility of being used in a daily menu.

The fruiting bodies of the fungus *Macrolepiota procera* are the raw material that meets the requirements of healthy nutrition. They are valued for their high protein content, rich mineral composition, and low fat content. They are considered low in calories, and due to their delicate taste and aroma, they are an alternative to meat. When analyzing the advantages of dishes containing *M. procera*, their health-promoting effect on the human body cannot be overlooked: they contain many bioactive substances with a very wide range of positive effects. Dishes and products containing the fruiting bodies of this species are an element-supporting therapeutic processes (e.g., in the treatment and prevention of depression, in antibiotic therapy, or in the fight against disturbances in the composition of the intestinal microflora), as well as with a prophylactic effect (e.g., used to delay changes caused by aging cells or reduce the risk of cancer). The cultivation of these mushrooms under controlled conditions may additionally enrich the mushroom raw material with the selected minerals and other ingredients. For these reasons, these mushrooms should be permanently indicated on the menu.

In order to reduce the loss of bioactive substances with antioxidant and antidepressant properties, this raw material should be processed briefly: it is better to fry briefly in breadcrumbs (like chops) or without breading than to prepare soups from finely chopped pieces of the fruiting bodies. An interesting alternative would be to use these mushrooms as an additional raw material or seasoning for the production of meatballs or delicatessen products (rolls, pates, tarts, or casseroles). This ingredient would enrich products with fiber, minerals, and bioactive ingredients, as well as reduce the fat content. Moreover, due to the presence of bioactive compounds in mushrooms, the finished products would have a prophylactic and therapeutic effect on consumers.

## Figures and Tables

**Table 1:** Production of cultivated mushrooms in the world in 2018-2020 (data according to FAOSTAT [17]).

Country	Production volume (tons)			Participation in world production (%)		
	2018	2019	2020	2018	2019	2020
China	37,890,000	38,950,652	40,000,000	93.32	93.33	93.5
Japan	467,000	470,000	471,810	1.15	1.27	1.10
USA	416,050	383,960	370,280	1.02	0.92	0.86
Poland	200,160	234,700	182,900	0.49	0.56	0.43
Spain	166,250	170,160	166,010	0.41	0.41	0.39
Canada	125,565	132,114	132,589	0.31	0.32	0.31
United Kingdom	98,509	101,339	105,660	0.24	0.24	0.25
France	82,980	87,560	80,010	0.20	0.21	0.19
Germany	73,230	71,790	78,730	0.18	0.17	0.18
Italy	70,670	70,860	69,210	0.17	0.17	0.16
World	40,600,043	41,736,063	42,792 893	100	100	100

**Table 2:** The main components of the *Macrolepiota procera.*

Group	Compound	Source of information about presence of component	Structure^∗^	Molecular formula	Molecular weight (g/mol)	Color (and form)	Source of information about properties of component
Carbohydrates	Mannitol (hexitol)	[39, 48–50]	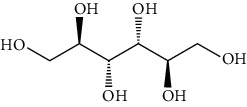	C_6_H_14_O_6_	182.17	White crystalline powder	[159, 160]
	Trehalose	[39, 46–50]	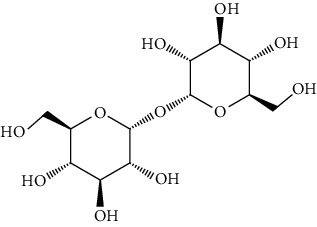	C_12_H_22_O_11_	342.30	White crystalline powder	[159, 160]
	Glycerol (glycerin)	[40]	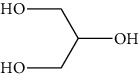	C_3_H_8_O_3_	92.09	Colorless syrupy liquid	[159, 160]
	Glucose	[40, 49]	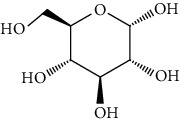	C_6_H_12_O_6_	180.16	Colorless crystals	[159, 160]
	Chitin	[40, 46, 47]	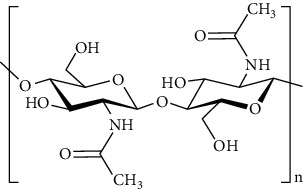	(C_8_H_13_NO_5_)n	203.19	Powder, usually from yellow to beige	[162]

Amino acids	Alanine	[53, 54]	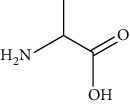	C_3_H_7_NO_2_	89.09	White crystalline powder	[159, 160]
	Proline	[53, 54]	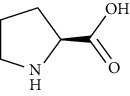	C_5_H_9_NO_2_	115.13	White crystals or crystalline powder	[159, 160]
	Glutamic acid	[53, 54]	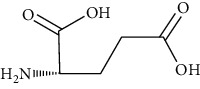	C_5_H_9_NO_4_	147.13	White crystalline powder	[159, 160]
	Serine	[53, 54]	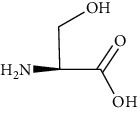	C_3_H_7_NO_3_	105.09	Solid	[159, 160]
Fatty acids	Linoleic acid	[39, 40, 53, 54]	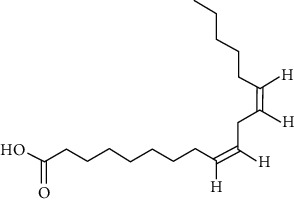	C_18_H_32_O_2_	280.45	Colorless, oily liquid	[160]
	Oleic acid	[39, 40, 53, 54]	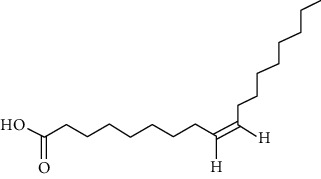	C_18_H_34_O_2_	282.46	Colorless, oily liquid	[160]
	Palmitic acid	[39, 40, 53, 54]	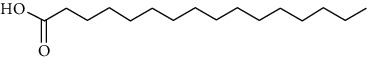	C_16_H_32_O_2_	256.42	White crystalline scales or needles	[160]

Fenolic acids	Caffeic acid	[101, 102, 134, 135]	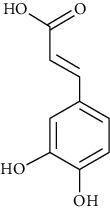	C_9_H_8_O_4_	180.16	Light yellow crystalline powder or yellow crystals	[159, 160]
	Cinnamic acid	[39, 80–82, 101–105, 133]	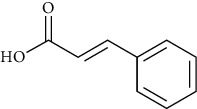	C_9_H_8_O_2_	148.17	Colorless crystals	[159, 160]
	p-Coumaric acid	[39, 80–82, 101, 133–135]	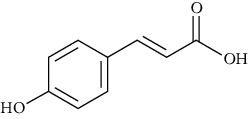	C_9_H_8_O_3_	16416.	Light yellow to beige crystalline powder	[161, 163]
	Ferulic acid	[82, 101, 134, 135]	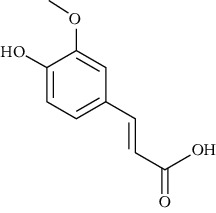	C_10_H_10_O_4_	194.18	Light yellow powder	[159, 160]
	Gallic acid	[82, 101–103, 134, 135]	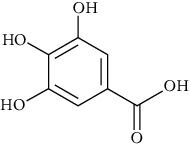	C_7_H_6_O_5_	170.12	White solid or powder	[159, 160]
	Gentisic acid	[101, 103]	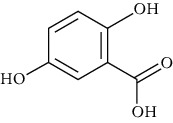	C_7_H_6_O_4_	154.12	White to yellow powder	[159, 160]
	4-Hydroxybenzoic acid (p-hydroxybenzoic acid)	[39, 80–82, 133]	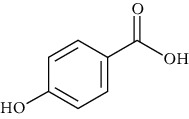	C_7_;H_6_O_3_	138.12	White to light beige crystalline powder	[159, 164, 165]
	Protocatechuic acid (3,4-dihydroxybenzoic acid)	[39, 80–82, 101–103, 105, 106, 133]	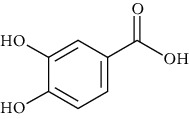	C_7_H_6_O_4_	154.12	White to light brown crystalline powder	[159, 160]
	Syringic acid	[82, 101, 103]	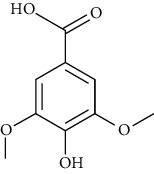	C_9_H_10_O_5_	198.17	Light brown powder	[159, 160]
	Tannic acid	[101]	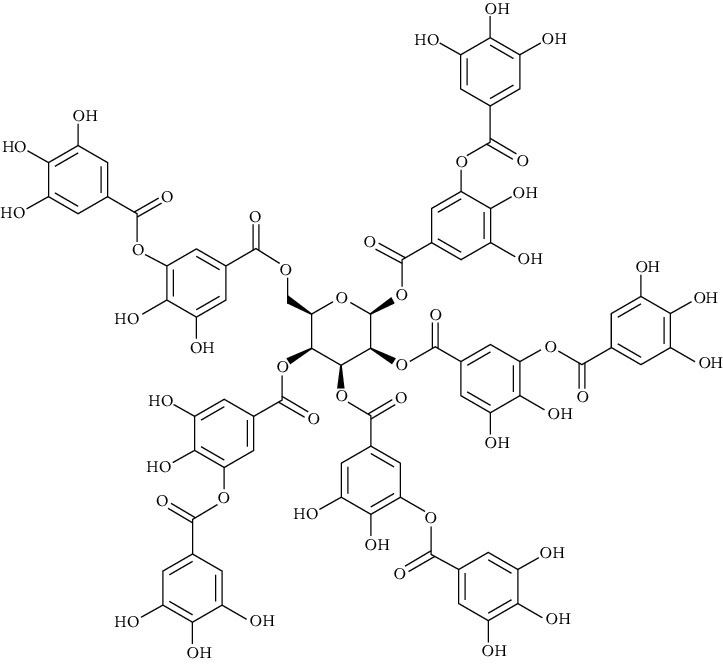	C_76_H_52_O_46_	1701.19	Light yellow to brown solid	[159, 160]
	Vanillic acid (4-hydroxy-3-methoxybenzoic acid)	[39, 81, 82, 101, 102]	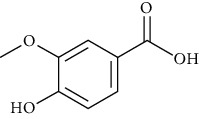	C_8_H_8_O_4_	168.15	White to light yellow crystals or powder	[159, 160]
Vitamins	Vitamin A (retinol)	[16]	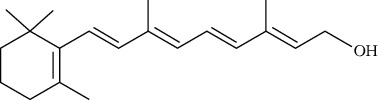	C_20_H_30_O	286.50	Oily liquid or yellow crystals	[159, 160]
	*α*-Tocopherol (vitamin E)	[50]	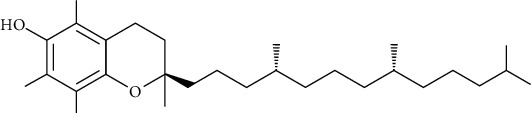	C_29_H_50_O_2_	430.71	Light yellow oil	[159]
	Ascorbic acid (vitamin C)	[42, 82, 83]	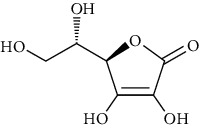	C_6_H_8_O_6_	176.13	White crystalline powder or colorless crystals	[159, 160]

Fruit acids	Malic acid	[42, 133]	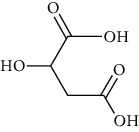	C_4_H_6_O_5_	134.09	White crystalline powder	[159, 160]
	Citric acid	[42, 133]	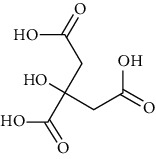	C_6_H_8_O_7_	192.12	White (or colorless) crystalline solid	[160]

Carotenoids	*β*-Carotene	[43, 82, 83, 98, 100, 119]	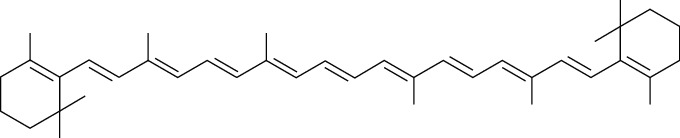	C_40_H_56_	536.87	Red to brownish-red crystals or crystalline powder	[159, 160]
	Lycopene	[43, 82, 83, 98, 100, 119]	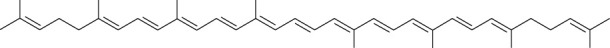	C_40_H_56_	536.87	Dark red solid	[159, 160]

Indole and tryptophan derivatives	Indole	[107]	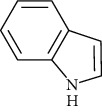	C_8_H_7_N	117.15	White crystals	[159, 160]
	L-Tryptophan	[107, 157]	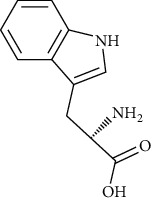	C_11_H_12_N_2_O_2_	204.22	Yellowish-white crystals or powder	[159, 160]
	5-Hydroxytryptophan	[50, 107, 157]	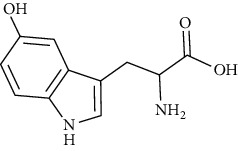	C_11_H_12_N_2_O_3_	220.22	n.d.	[159, 160]
	5-Methyltryptamine	[107]	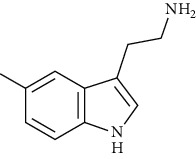	C_11_H_14_N_2_	174.24	n.d.	[159, 160]
	Melatonin	[107, 157]	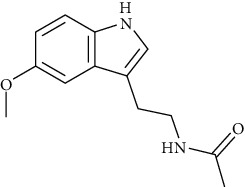	C_13_H_16_N_2_O_2_	232.28	White-cream to yellowish crystalline powder	[159, 160]
	Tryptamine	[107, 157]	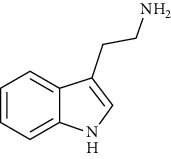	C_10_H_12_N_2_	160.22	Slight-yellow or pale-white crystalline powder	[159, 160]

^∗^Structural formulas are derived from the source of information about the properties of the component.

**Table 3:** Studies of antioxidant properties of substances present in the *Macrolepiota procera* fruiting bodies.

Type of test^∗^	Type of extracts^∗^	Parameter determined	Results	Authors
(i) Total phenolic content (ii) *β*-carotene content (iii) Lycopene content (iv) DPPH^∗∗^	M	(i) Absorbance at 760 nm (ii) Absorbance at 453, 505, and 663 nm (iii) Absorbance at 453, 505, and 663 nm (iv) Absorbance at 517 nm (IC_50_)	(i) 36.25 ± 0.35 mg GAE/g extract (ii) 0.091 ± 0.09 *μ*g/ml (iii) 0.059 ± 0.02 *μ*g/ml (iv) IC_50_ = 0.191 ± 0.07 mg/ml	[100]
(i) Reducing power (a) Folin-Ciocalteu (b) Ferricyanide/Prussian blue (ii) Radical scavenging activity (a) DPPH (b) *β*-Carotene/linoleate (iii) Lipid peroxidation inhibition (a) Thiobarbituric acid reactive substance (TBARS)	M	(i) n.d. (percentage of activity) (ii) Absorbance at 690 nm (EC_50_) (iii) n.d. (percentage of activity; EC_50_) (iv) Linoleate assay (EC_50_) (v) TBARS assay (EC_50_)	(i) 13.9 ± 0.6 mg GAE per g extract (ii) EC_50_ = 1.61 ± 0.01 mg ml^−1^ (iii) EC_50_ = 3.7 ± 0.2 mg ml^−1^ (iv) EC_50_ = 0.48 ± 0.04 mg ml^−1^ (v) EC_50_ = 0.27 ± 0.02 mg ml^−1^	[123]
(i) Total phenolic content (ii) FRAP	M	(i) Absorbance at 760 nm (ii) Absorbance at 593 nm	(i) TPC = 1.624 ± 0.026 mg GAE g^−1^ (ii) FRAP = 4.245 ± 0.042 *μ*mol FeSO_4_ · 7H_2_O g^−1^	[44]
(i) DPPH (ii) Reducing power (iii) Total phenolic content	M	(i) Absorbance at 517 nm (ii) Absorbance at 700 nm (iii) Absorbance at 760 nm	(i) IC_50_ = 311.40 ± 1.28 *μ*g/ml (ii) 1000 *μ*g/ml: 0.9001 ± 0.043; 500 *μ*g/ml: 0.4453 ± 0.030; 250 *μ*g/ml: 0.3722 ± 0.012; 125 *μ*g/ml: 0.1182 ± 0.009 (iii) TPC = 67.98 ± 1.013 *μ*g PE/mg of extract	[54]
(i) DPPH (ii) Lycopene and carotene antioxidant activity assays (iii) Total phenolic compounds (iv) Total flavonoid (v) Ferrous ion chelating assay (percentage of activity; EC_50_)	M	(i) Absorbance at 515 nm (DPPH radical; percentage of activity; EC_50_) (ii) Absorbance at 453, 505, and 663 nm (iii) Absorbance at 515 nm (Folin-Ciocalteu reagent and sodium carbonate) (iv) Absorbance at 515 nm (aluminum nitrate and aqueous potassium acetate) (v) Absorbance at 562 nm	(i) Activity: 65.41%; EC_50_ = 0.24 mg ml^−1^ (ii) Lycopene: 5.37 ± 0.55 100 g^−1^; *β*-carotene: 11.57 ± 2.39 mg 100 g ^−1^ (iii) TPC = 136.21 ± 0.98 mg GAE 100 g^−1^ (iv) TF = 8.66 ± 1.08 mg QE 100 g^−1^ (v) Activity: 91.45%; EC_50_ = 0.13 mg ml^−1^	[119]
(i) FRAP	M	(i) Absorbance at 595 nm	1.8 mmol FE^2+^/100 g fw	[52]
(i) Trolox equivalent antioxidative activity (TEAC) measurement (ii) Reducing power (iii) Assay for total phenolics (iv) Assay for total flavonoids	M, A	(i) Absorbance at 414 nm (ii) Absorbance at 700 nm (iii) Absorbance at 765 nm (iv) Absorbance at 515 nm	(i) Cap—M: 8.92 ± 0.04; A: 36.08 ± 0.37; stalk—M: 5.09 ± 0.89; A: 21.83 ± 3.13 (*μ*mol of Trolox/g dw) (ii) Cap—M: 2.25 ± 0.11; A: 8.99 ± 0.25; stalk—M: 2.90 ± 0.08; A: 9.64 ± 0.29 (*μ*g of gallic acid/g dw) (iii) Cap—M: 2.17 ± 0.39; A: 10.30 ± 1.50; stalk—M: 1.95 ± 0.30; A: 7.51 ± 0.50 (*μ*g of gallic acid equivalents/mg dw) (iv) Cap—M: 0.918 ± 0.37; A: 5.13 ± 0.07; stalk—M: 0.75 ± 0.04; A: 2.18 ± 0.03 (*μ*g of quercetin equivalents/mg dw)	[98]
(i) DPPH (ii) Total phenolic content	A	(i) Absorbance at 517 nm (IC_50_) (ii) Absorbance at 765 nm	(i) RSA = 88.1 ± 2.1%; AAE = 191.00 ± 43.46 mg · l^−1^; IC_50_ = 0.948 mg (ii) TPC = 2.429 ± 0.119 g · kg^−1^	[118]
(i) Total phenolic content (ii) DPPH (iii) ABTS+radical cation scavenging assay (iv) Reducing power	A	(i) Absorbance at 765 nm (ii) Absorbance at 517 nm (EC_50_) (iii) Absorbance at 734 nm (EC_50_) (iv) Absorbance at 700 nm (EC_50_)	(i) Sun-drying: 0.7658 ± 0.04366; freeze drying: 1.2329 ± 0.0556 (g% tannic acid) (ii) Sun-drying: 5.0019 ± 0.0821; freeze drying: 2.4962 ± 0.0198 (iii) Sun-drying: 8.7461 ± 1.8188; freeze drying: 4.9284 ± 0.1447 (iv) Sun-drying: 5.3204 ± 0.1202; freeze drying: 1.0867 ± 0.1320	[97]
(i) Hydroxyl radical scavenging activity (ii) Superoxide radical scavenging activity (iii) *β*-Carotene bleaching assay	AMd	(i) Absorbance at 535 nm (EC_50_) (ii) Absorbance at 560 nm (EC_50_) (iii) Absorbance at 490 nm (EC_50_)	(i) EC_50_ = 875 *μ*g/ml (ii) EC_50_ = 80 *μ*g/ml (iii) EC_50_ = 345 *μ*g/ml	[120]

^∗^Type of extracts: M: methanolic extract; A: aqueous extract; AMd: aqueous extract of fruit bodies *M. dolichaula*. ^∗∗^Type of test: DPPH: 1-diphenyl-2-picrylhydrazyl (DPPH) radical scavenging activity; FRAP: ferric reducing/antioxidant power assay; dw: dry weight; fw: fresh weight.

**Table 4:** Studies on the use of substances contained in *Macrolepiota procera* for medical purposes (from 2015 to 2021).

Substances	Test organism	Type of test/parameter	Concentration/dose	Authors
Methanolic extract	*In vitro*: HeLa cells (human epithelial carcinoma), A549 cells (human lung carcinoma), LS174 cells (human colon carcinoma)	Cytotoxic assay (absorbance at 570 nm), IC_50_	12.5 mg/ml, 25 mg/ml, 50 mg/ml, 100 mg/ml, 200 mg/ml	[54]
	*In vitro*: bacteria *Bacillus cereus* (ATCC 10987), *B. subtilis* (ATCC 66330), *Escherichia coli* (ATCC 25922), *Proteus mirabilis* (ATCC 12453), *Staphylococcus aureus* (ATCC 25923); fungi *Alternaria alternata* (ATCC 36376), *Aspergillus flavus* (ATCC 9170), *A. niger* (ATCC 16888), *Candida albicans* (ATCC 10259), *Cladosporium cladosporioides* (ATCC 11680), *Fusarium oxysporum* (ATCC 62506), *Mucor mucedo* (ATCC 20094), *Penicillium chrysogenum* (ATCC 10106), *P. expansum* (ATCC 20466), *Trichoderma viride* (ATCC 13233)	Minimal inhibitory concentration (MIC)	0.0195–40 mg/ml	
Lepiotapro-cerins A-L	*In vitro*: murine monocytic RAW 264.7 macrophages	Nitric oxide production in RAW 264.7 macrophages (absorbance at 570 nm)	Dilutions up to the maximum concentration 25 *μ*M	[112]
	*In vitro*: human myeloid leukemia (HL-60; ATCC CCL-240), lung cancer (A-549; ATCC CCL-185), human hepatocellular carcinoma (SMMC-7721), breast cancer (MCF-7; ATCC HTB-22), human colon cancer (SW480; ATCC CCL-228)	Cytotoxicities against cancer cell lines (assess cell viability; absorbance at 595 nm), IC_50_		
	*In vitro*: *Mycobacterium tuberculosis* H37Ra	Antimycobacterial assay (green fluorescent protein microplate assay)	n.d.^∗^	
Methanolic extract	*In vitro*: bacteria *Bacillus cereus* NRRL B-3711, *B. subtilis* ATCC 6633, *Escherichia coli* ATCC 35218, *E. coli* ATCC 25922, *Enterococcus faecalis* ATCC 29212, *E. hirae* ATCC 9790, *Klebsiella pneumaniae* ATCC 13883, *Pseudomonas aeruginosa* ATCC 27853, *Proteus vulgaris* RSKK 96029, *Salmonella typhimurium* ATCC 14028, *Staphylococcus aureus* ATCC 25923; fungi *Candida albicans* ATCC 10231, *C. krusei* ATCC 6258, C*. tropicalis* Y-12968	Antimicrobial activity (diameter of the inhibition zones)	150 mg/ml	[100]
Aqueous extract	*Pseudomonas aeruginosa* 44, *P. aeruginosa* 119	Microtitre plate method (inhibition of bacterial activity)	50 mg/ml	[118]
Methanolic extract	*In vitro*: *Acinetobacter haemolyticus* ATCC 19002, *Enterococcus faecalis* ATCC 29212, *Escherichia coli* ATCC 25922, *Klebsiella pneumoniae* ATCC 13883, *Proteus mirabilis* ATCC 7002, *Pseudomonas aeruginosa* ATCC 27853, *Salmonella typhimurium* ATCC 14028, *Staphylococcus aureus* ATCC 25923	Agar well diffusion method	100 mg/ml	[44]
Methanolic extracts	*In vivo*: mice	Suppressive effect against tumor promoter-induced inflammation (by TPA)	n.d.	[157]
Macrospin (trypsin inhibitors)	*In vitro*: trypsin and chymotrypsin	Protease inhibition assays	0.11 *μ*M-85 mM	[156]
Fructo-galactan PS II	(i) *In vitro*: RAW 264.7 (murine macrophage cell line) (ii) Suspension of the spleen and thymus cell (from mice)	(i) Content of NO (absorbance at 540 nm) (ii) Standard MTT assay method (splenocyte proliferation index and thymocyte proliferation index)	12.5, 25, 50, 100, 200 *μ*g/ml	[120]
Chloroform-acetone extract of Mk^∗^	Bacteria: *Bacillus cereus*, *B. athrophaeus*, *Escherichia coli*, *Micrococcus luteus*, *Staphylococcus aureus*, *S. epidermidis*; fungi: *Aspergillus clavatus*, *A. niger*, *Botrytis cinerea*, *Mucor* sp., *Penicillium chrysogenum*, *Rhizopus* sp.	Well diffusion method	200 mg ml^−1^	[154]

^∗^Symbols: n.d.: no date; Mk: *Macrolepiota konradii.*

**Table 5:** Action mechanisms by bioactive substances contained in *Macrolepiota procera*, other than antioxidant (from 2015 to 2021).

Activity	Result of mechanism	Mechanism	Authors
Antitumor effect	(i) Inhibition of the development of cancer cells (HeLa, A549, LS174)	(i) n.d.^∗^	[54]
	(i) Inhibitory effect on the activity of tumor cells A-549, HL-60, MCF-7, SMMC-7721, SW-480	(i) n.d.	[112]
Antibacterial effect	(i) Inhibition of activity of bacteria Bacillus cereus, B. subtilis and Proteus mirabilis and fungi Alternaria alternata, Aspergillus niger, Candida albicans, Fusarium oxysporum, Penicillium expansum, P. chrysogenum, and Trichoderma viride	(i) n.d.	[54]
	(i) Inhibition of colony development of *Enterococcus faecalis* ATCC 29212 and *Klebsiella pneumaniae* ATCC 13883	(i) n.d.	[100]
	(ii) High anti-QS activity against *Pseudomonas aeruginosa* 44 and. *P. aeruginosa* 119 (iii) No effect on bacterial growth	(i) n.d.	[118]
	(i) Inhibition of the activity of *Mycobacterium tuberculosis* H37Ra	(i) n.d.	[112]
	(ii) Inhibitory effect against *Klebsiella pneumoniae* and *Escherichia coli*	(ii) Inhibition of the growth of bacterial colonies (cell division)	[44]
	(iii) Inhibition of the life activity of bacteria (Gram+: *S. aureus*, *S. epidermidis*, *M. luteus* and Gram-: *E. coli*)	(iii) Decrease in the viability of bacterial cells	[154]
Anti-inflammatory effect	(i) Inhibitory effect on induced inflammation	(i) Suppression of the TPA effect (swelling inhibition)	[157]
	(ii) Inhibition of inflammation	(ii) Inhibition of NO production in RAW 264.7 macrophages	[112]
Regulating effect	(i) Strong inhibition of trypsin and weaker of chymotrypsin	(i) n.d.	[156]
Immunostimulating effect	(i) Activation of macrophages (ii) Splenocyte and thymocyte stimulator	(i) Increase in NO production (ii) Stimulation of the proliferation of thymocytes and splenocytes (increase in proliferation index)	[120]
Antidepressant effect	(i) Increase in serotonin and melatonin levels	(i) Presence of L-tryptophan, 5-hydroxytryptophan, tryptamine, serotonin, and melatonin—precursors and neurotransmitters with antidepressant properties (ii) Conversion of precursors into neurotransmitters	[107, 157]

^∗^Symbols: n.d.: no data (not tested).
